# Enhancement of CD117-Targeted Bispecific T-cell Engagement by CD33-Targeted Bispecific T-cell Costimulation in Acute Myeloid Leukemia

**DOI:** 10.1158/2767-9764.CRC-25-0672

**Published:** 2026-04-27

**Authors:** Mara Hofstetter, Laura Volta, Christian Koch, Melanie Granados Rey, Monique Maurer, Nagihan Gönüllü, Christian Pellegrino, Celeste Gobbi, Florin Schneiter, Francesco Manfredi, Chiara F. Magnani, Abdullah Elsayed, Timm Schroeder, Dario Neri, Markus G. Manz

**Affiliations:** 1 Department of Medical Oncology and Hematology, University and University Hospital Zürich, Zürich, Switzerland.; 2 Comprehensive Cancer Center Zürich, Zürich, Switzerland.; 3 Department of Biosystems Science and Engineering, ETH Zurich, Basel, Switzerland.; 4 Philochem AG, Otelfingen, Switzerland.; 5 Department of Chemistry and Applied Biosciences, Institute of Pharmaceutical Sciences, Swiss Federal Institute of Technology (ETH Zürich), Zürich, Switzerland.

## Abstract

Acute myeloid leukemia (AML) originates from the hematopoietic stem and progenitor cell compartment and is associated with an overall poor clinical outcome. T cell–engaging bispecific antibodies (TCE), binding to a tumor-associated antigen and to CD3, redirect T cells to target antigen–expressing cells in an MHC/TCR-independent fashion. The development of TCEs for clinical application is facing several challenges. First, it is difficult to identify a tumor-selective, non–MHC-presented tumor target, not expressed on the tissue of tumor origin, thus limiting specificity. Second, CD3-directed TCEs do not provide a second, T cell–activating signal, such as the stimulation of CD28 or 41BB, thus possibly limiting T-cell efficacy. To address both aspects, we generated a CD33xCD28 IgG4-scFv_2_ with CD28 agonistic activity and tested it in combination with a previously by us reported CD117xCD3 TCE in AML. Combining these two bispecific antibodies significantly improved T cell–mediated lysis of AML cell lines and primary AML samples by enhancing T-cell activation, proliferation, and cytokine release *in vitro*. Furthermore, the addition of CD33xCD28 IgG4-scFv_2_ showed faster time to cell attachment, increased lytic events, and improved specificity toward double-target–expressing cells. In summary, the data indicate that combining costimulation via a second tumor-associated antigen to CD3-TCEs enhances T-cell lytic activity and simultaneously increases specificity against double-target–expressing AML cells.

**Significance:**

Current TCEs lack costimulatory signaling. The here-described CD33xCD28 IgG4-scFv_2_ delivers target-selective costimulation and enhances activation, proliferation, cytokine release, and cytotoxicity when used in combination with CD117xCD3 TCE in AML *in vitro*. Furthermore, the dual tumor-associated antigen targeting improves therapeutic specificity by preferentially eliminating CD117^+^CD33^+^ cells, possibly allowing improved bispecific antibody–mediated AML therapy.

## Introduction

Acute myeloid leukemia (AML) is a hematopoietic malignancy characterized by the accumulation of genetic mutations that lead to uncontrolled proliferation and impaired differentiation of hematopoietic stem and progenitor cells (HSPC), ultimately resulting in healthy bone marrow failure (1–3).

Current therapeutic strategies for younger and fit patients typically involve intensive chemotherapy followed possibly by hematopoietic stem and immune cell transplantation (HSCT). In contrast, elderly and frail patients are mostly treated with low-intensity regimens such as hypomethylating agents and BCL-2 inhibitors (4–6). Although many patients initially respond to therapy, relapse is common, with long-term survival rates of approximately 30% to 45% in younger patients and only 10% to 15% in older individuals (7–9). These outcomes underscore the need for novel, more efficient therapeutic approaches.

T cells play a central role in antitumor immunity. Upon engagement of the T-cell receptor (TCR) with a peptide–MHC complex on antigen-presenting cells (signal 1), full T-cell activation requires a second co-stimulatory signal (signal 2), typically mediated by costimulatory receptors, such as CD28, on T cells interacting with ligands, like CD80/CD86, on antigen-presenting cells. Integration of both signals is critical for robust T-cell activation, clonal expansion, cytokine secretion, and effective tumor cell lysis (10–13).

The importance of costimulation also became evident in chimeric antigen receptor (CAR) T-cell therapy, in which second-generation CAR T cells incorporating both CD3ζ and costimulatory domains (e.g., CD28 or 4-1BB) outperformed first-generation CAR T cells lacking costimulatory signaling, in terms of activation, cytotoxicity, and persistence (14–16).

T cell–engaging antibodies (TCE) are a relatively new class of engineered antibody constructs, which allow redirection of T cells to target cells in an MHC/TCR-independent fashion by simultaneously binding to CD3ε on T cells and an antigen on target cells, e.g., a tumor-associated antigen (TAA) on malignant cells (17, 18). Several CD3-bispecific antibodies have been approved for hematologic malignancies, including blinatumomab (CD19xCD3), teclistamab (BCMAxCD3), mosunetuzumab, epcoritamab, and glofitamab (all CD20xCD3), which simulate activation of signal 1 and induce tumor cell lysis (19). To enhance efficacy, bispecific antibodies targeting a second TAA for local delivery of signal 2 via an agonistic costimulatory molecule binder are being explored in B-cell and plasma cell malignancies. Indeed, enhanced efficacy was demonstrated in combinatorial settings of signals 1 and 2 (20–22).

We hypothesized that activating T cells via CD3 and CD28, mediated by two bispecific binders to two AML-associated antigens, would enhance overall therapeutic efficacy. Moreover, given the heterogeneity of AML, dual TAA targeting might also improve specificity, a concept which has been supported by previous studies (23–28). We therefore in this study investigate the combination of our previously described CD117xCD3 TCE (29), targeting the receptor for stem cell factor (SCF), being expressed on HSCs and leukemia initiating cells, with a newly generated CD33xCD28 IgG-scFv_2_. This bispecific construct targets the more broadly myelopoiesis and AML expressed antigen CD33, a cell surface receptor belonging to the sialic acid–binding immunoglobulin-like lectin family (30–32), a well-known target-antigen for immunotoxins in clinical use (33).

## Materials and Methods

### Cell lines

All cell lines were authenticated and tested for *Mycoplasma* by the provider. No further *Mycoplasma* testing or cell line authentication was performed. Cells were kept in culture for a maximum of 2 months and used in experiments after 1 week of thawing. HL-60 (ATCC, RRID: CVCL_0002, derived from a female with acute promyelocytic leukemia and purchased in 2019), KASUMI-1 (ATCC, RRID: CVCL_0589, derived from a male AML patient and purchased in 2019), and MOLM-14 (DSMZ, RRID: CVCL_7916, derived from a male AML patient and purchased in 2020) were cultured in RPMI 1640 supplemented with 10% fetal bovine serum (FBS) and 1% penicillin/streptomycin (all obtained from Gibco, Thermo Fischer Scientific, cat. #11875093, A5256801, 15140122) at 37°C in a humidified atmosphere containing 5% CO_2_. Both MOLM-14 and HL-60 cells were previously transduced via lentiviral vectors to express the truncated GNNK^+^ isoform of human CD117 (c-Kit), along with a dual reporter system (GFP/Luciferase), enabling the expression of low, intermediate, and high levels of CD117 (34). MOLM-14 CD33 knockout (CD33KO) cells were kindly provided by Prof. Dr. Saar Gill (University of Pennsylvania) and similarly transduced to express CD117 (25).

Bispecific antibodies were produced in Chinese Hamster Ovary cells (CHO-S, RRID: CVCL_7183), maintained in PowerCHO medium (Lonza, cat. #BELN12-771Q) supplemented with 8 mmol/L GlutaMAX, 4 mmol/L HT supplement, and 1% antibiotic–antimycotic (all from Gibco, Thermo Fischer Scientific, cat. #35050-038, 41065-012, 15240-062). For protein production, CHO-S cells were transferred to ProCHO-4 medium (Lonza, cat. #BEBP12-029Q) with identical supplementation. Cultures were maintained in a shaking incubator at 37°C for maintenance, 31°C for protein production, and 5% CO_2_ in a humidified atmosphere.

### Primary human cells

Patient and healthy donor data and cells were obtained from the Department of Medical Oncology and Hematology Biobank, University Hospital Zürich, Zürich, Switzerland, and written informed consent was obtained from all patients. This study was conducted in accordance with the Declaration of Helsinki and approved by the Cantonal Ethics Board of Zürich, Switzerland (2009-0062).

#### Healthy donor–derived peripheral blood mononuclear cells and T cells

Buffy coats were obtained from the Zürich Blood Donation Service (Blutspende Zürich). All donor samples are anonymized. The blood type is the only donor information provided and recorded. Peripheral blood mononuclear cells (PBMC) were isolated via density gradient centrifugation using Ficoll-Paque Plus (Cytiva, cat. #17144003). When human T cells were required, negative selection was performed using the EasySep Human T-Cell Isolation Kit (STEMCELL Technologies, cat. #17951). PBMCs and T cells were cryopreserved in liquid nitrogen in FBS containing 10% dimethyl sulfoxide (DMSO) until further use. Flow cytometric phenotyping was conducted to assess immune cell populations: PBMCs were analyzed for hCD19, hCD3, hCD4, and hCD8 expression, whereas T cells were evaluated for hCD3, hCD4, and hCD8 markers (see Supplementary Table S1).

#### Primary AML cells

Peripheral blood samples from patients diagnosed with AML were collected at the Department of Medical Oncology and Hematology, Zürich (Switzerland), at diagnosis upon written informed consent from participants. The study was conducted in accordance with the Declaration of Helsinki and approved by the Cantonal Ethics Board Zürich (approval number: 2009-0062, Switzerland). Mononuclear cells were isolated via density gradient centrifugation. In selected cases, a CD3^+^/CD19^+^ double depletion was performed using human CD3 and CD19 MicroBeads (Miltenyi Biotec, cat. #130-136-718), following the manufacturer’s protocol. The resulting cell populations were collected and cryopreserved as previously described (35). Detailed characteristics of the AML patient samples used in this study are provided in Supplementary Table S2.

### Protein development and production

#### CD117xCD3 TCE

The CD117xCD3 TCE is a tandem single-chain variable fragment (scFv) comprising two antigen-binding domains: one targeting CD117 (clone 79D) and the other targeting CD3 (clone OKT3), connected via a flexible GGGGS linker. The construct was cloned into the pcDNA3.1(+) expression vector (Invitrogen, Thermo Fisher Scientific, cat. #V79020) and produced as previously described (29).

#### CD33xCD28 IgG4-scFv_2_

The pMM137 plasmid, containing a mammalian secretion signal and the CD28 (clone E1P2) IgG4 sequence, was kindly provided by Philochem/Philogen and previously described (36).

The CD33 (SGN-33) sequence previously published (37) was cloned into the plasmid as a scFv at the C-terminus via a short glycine–serine (GS) linker.

Plasmid transformation and amplification were performed in *Escherichia coli* DH5α cells (Thermo Fischer Scientific, cat. #18258012), followed by plasmid isolation using the NucleoBond Xtra Maxi Plus Kit (Macherey-Nagel, cat. #740416.50). For protein expression, CHO-S cells were resuspended at 4 × 10^6^ cells/mL in supplemented ProCHO-4 medium and transfected to induce transient gene expression (TGE). Transfection was carried out by adding 3.2 mg/L of plasmid DNA and 10 mg/L of polyethylenimine (Polysciences, Inc., cat. #23966-1). Cultures were maintained in a humidified shaking incubator at 31°C and 5% CO_2_ for 6 days.

After incubation, the supernatant was harvested and subjected to protein A affinity chromatography using UNOsphere SUPra media (Bio-Rad, cat. #1560218) overnight at 4°C. Elution was performed with an in-house prepared 0.1 mol/L glycine buffer (pH 3), and buffer exchange into phosphate-buffered saline (PBS; Gibco, Thermo Fischer Scientific, cat. #10010023) was conducted using PD-10 columns (Cytiva, cat. #17085101). The final formulation was supplemented with 0.02% Tween-80 (Merck, cat. #P1754).

Further purification was achieved via fast protein liquid chromatography (RRID: SCR_023461, Cytiva) using a Superdex 200 Increase 10/300 GL column (Cytiva, cat. #28990944) at a flow rate of 0.75 mL/minute.

Protein integrity and purity were evaluated by SDS-PAGE, size-exclusion chromatography, and liquid chromatography–mass spectrometry (LC-MS). Size-exclusion chromatography was performed using the aforementioned column and flow rate. Peak areas were used to quantify the proportion of each species. LC-MS analysis was conducted by the Functional Genomics Center Zurich of the University of Zurich and ETH Zurich on a Waters Xevo G2-XS QTof instrument (ESI-ToF-MS) coupled to a Waters Acquity UPLC H-Class System, equipped with a 2.1 × 50 mm Acquity BEH300 C4 1.7 μm column (Waters), using solvents as previously described (38).

### Flow cytometry–based assays

Dead cells were identified using one of the following viability dyes: Zombie Aqua (BioLegend, cat. #423107), Zombie Violet (BioLegend, cat. #423113), or Hoechst 33342 (Thermo Fischer Scientific, cat. #H3570). When Zombie Aqua or Zombie Violet was used, cells were first incubated with the viability dye in PBS for 15 minutes at 4°C. This was followed by staining with fluorescently labeled antibodies (see respective combinations in Supplementary Table S1) in FACS buffer (PBS supplemented with 2% FBS and 2 mmol/L EDTA, PanReac AppliChem cat. #A4892,0100) for 30 minutes at 4°C.

If Hoechst 33342 was used, cells were first stained with fluorescently labeled antibodies as described above. Hoechst 33342 was then added before acquisition on the flow cytometer (1:2,000 in PBS).

Samples were acquired using a BD LSR Fortessa II flow cytometer (Becton Dickinson) and analyzed with FlowJo software (v10.0.7, Treestar, RRID: SCR_008520).

### Binding assay

MOLM-14 CD117^High^GFP^+^Luc^+^ cells were coincubated in a 96-well plate with a titration of CD33xCD28 IgG4-scFv_2_ for 1 hour at 4°C. After washing, it was incubated with the secondary antibody goat anti-human IgG (see Supplementary Table S1) for 20 minutes at 4°C and analyzed by flow cytometry.

### Safety assay

Flat-bottom plates (96-well) were coated with PBS containing 10 μg/mL of either CD28 TGN1412 provided by Philochem/Philogen, CD28 IgG4, CD33xCD28 IgG4-scFv_2_, CD117xCD3, or combinations of CD117xCD3 with either CD28 IgG4 or CD33xCD28 IgG4-scFv_2_. Coating was performed for 2 hours in a humidified incubator at 37°C and 5% CO_2_. PBS served as a negative control. After careful removal of the coating solution, 1 × 10^5^ PBMCs derived from healthy donors were plated in each well. Cells were cultured in T-cell medium, comprising Advanced RPMI 1640 medium (Gibco, Thermo Fischer Scientific, cat. #12633012) supplemented with 10% FBS, 1% penicillin/streptomycin, and 1% GlutaMAX. Following a 96-hour incubation under the same conditions, supernatants were harvested and stored at −20°C for subsequent cytokine analysis. Cells were stained according to the markers listed in Supplementary Table S1 and analyzed by flow cytometry using a high-throughput screening (HTS) platform.

### *In vitro* cytotoxicity assays

#### Cytotoxicity assay with AML cell lines

Cocultures of AML cell lines with healthy donor–derived T cells were established at an effector-to-target (E:T) ratio of 1:1. Cultures were treated with a serial dilution of CD117xCD3 alone or in combination with 0.025, 0.5, 1, or 10 nmol/L of either CD28 IgG4 or CD33xCD28 IgG4-scFv_2_. Cells were plated in T-cell medium in 96-well round-bottom plates and incubated for 96 hours in a humidified incubator at 37°C and 5% CO_2_.

After incubation, supernatants were collected and stored at −20°C for subsequent cytokine analysis. Cells were stained with fluorescently labeled antibodies as listed in Supplementary Table S1. For experiments involving MOLM-14 cells, Hoechst 33342 and CountBright absolute counting beads (Invitrogen, cat. #C36950) were added immediately prior to flow cytometry acquisition. For HL-60 and KASUMI-1 cells, staining was performed in advance using Zombie Aqua, followed by antibody staining, and samples were directly acquired on the flow cytometer.

Specific lysis was calculated using the following formula:

$$Specific\;lysis\;=\;\left(1\;-\;\frac{Number\;of\;alive\;target\;cells}{Number\;of\;alive\;target\;cells\;in\;control\;group}\right)\;\times \;100$$

All experiments were performed using T cells from three independent healthy donors, each plated in duplicate.

#### Specificity assay

Healthy donor–derived T cells were cocultured with a mixed population of MOLM-14 cell lines at an E:T ratio of 1:1. The MOLM-14 mixture consisted of equal proportions of MOLM-14 CD117^High^CD33^+^, MOLM-14 wild-type (CD117^−^CD33^+^), and MOLM-14 CD117^High^CD33^−^ cells. Cultures were maintained in T-cell medium in 96-well round-bottom plates and treated with either CD117xCD3 alone or in combination with 0.5 nmol/L CD33xCD28 IgG4-scFv_2_.

At defined time points (4, 8, 12, 24, 48, 72, and 96 hours), cells were removed from the incubator and prepared for flow cytometry analysis. Staining was performed first with a live/dead marker, followed by fluorescently labeled antibodies as listed in Supplementary Table S1. Samples were acquired by HTS flow cytometry.

All experiments were conducted in duplicates using three different healthy donor–derived T cells.

Specific lysis was calculated using the following formula:

$$Specific\;lysis\;=\;\left(1\;-\;\frac{Number\;of\;alive\;cells\;of\;a\;specific\;subpopulation}{Number\;of\;alive\;cells\;of\;the\;specific\;subpopulation\;in\;control\;group\;}\right)\;\times \;100$$

#### Repetitive AML target cell lysis by T cells in combination with bispecific antibodies

MOLM-14 CD117^High^ cells were plated in 96-well round-bottom plates together with healthy donor–derived T cells at an E:T ratio of 1:1 in T-cell medium. Cocultures were treated either with 9.35 nmol/L CD117xCD3 TCE alone or in combination with 0.5 nmol/L CD33xCD28 IgG4-scFv_2_ and incubated at 37°C and 5% CO_2_ in a humidified incubator.

After 3 days, the cell-free supernatant was collected and stored at −20°C for cytokine analysis. Cells were stained for subsequent analysis with live/dead viability marker and fluorescently labeled antibodies listed in Supplementary Table S1, and acquisition was performed by HTS flow cytometry. Data were analyzed immediately to determine the remaining numbers of MOLM-14 CD117^High^ and T cells in each well to prepare the rechallenge.

Half of the conditions were selected for T-cell rechallenge, for which the number of MOLM-14 CD117^High^ cells to be added was calculated using the following formula:

MOLM14 to add = (average of T cells left of the same condition) - (average of MOLM14 left of the same condition)

The calculated number of MOLM-14 CD117^High^ cells and fresh CD117xCD3 TCE or the combination were added for the rechallenge wells. Plates were again incubated for 3 days in a humidified incubator at 37°C and 5% CO_2_. After this second incubation period, the cell-free supernatant was collected, and cells were stained for subsequent flow cytometry analysis.

#### Cytotoxicity assay with primary human AML samples

Primary human AML samples were thawed, transferred into tubes, and gradually resuspended in T-cell medium. Cells were then plated in 96-well round-bottom plates together with healthy donor–derived T cells at an approximate E:T ratio of 1:1 in T-cell medium.

Cultures were treated with CD117xCD3 TCE at concentrations of 18.7, 9.35, 4.675, or 0.94 nmol/L, either alone or in combination with 0.5 nmol/L CD28 IgG4 or CD33xCD28 IgG4-scFv_2_. Cocultures were incubated for 48 hours in a humidified incubator at 37°C and 5% CO_2_.

After incubation, cell-free supernatants were collected and stored at −20°C for subsequent cytokine analysis. Cells were stained with a live/dead viability marker, followed by fluorescently labeled antibodies (see Supplementary Table S1), and samples were acquired by HTS flow cytometry.

Additionally, patient samples were evaluated by flow cytometry for expression of human CD117 and CD33. All experiments were performed in duplicate using T cells from two independent healthy donors.

#### Enzyme-linked immunosorbent assay

Interferon γ (IFNγ) levels secreted by cells were quantified using enzyme-linked immunosorbent assay (ELISA) on supernatants collected from cocultures after incubation. The assay was performed according to the manufacturer’s protocol using the ELISA MAX Deluxe Set (BioLegend, cat. #430104). Cytokine concentrations were measured at an absorbance of 450 nm using a Mini ELISA Plate Reader (BioLegend, cat. #423555).

### Live-cell imaging

Time-lapse experiments were conducted at 37°C and 5% CO_2_ in RPMI 1640 growth medium supplemented with 2 μmol/L propidium iodide (eBioscience, cat. #BMS500PI), 10% FBS, and 1% penicillin/streptomycin using μ-slides with four wells (Ibidi, cat. #80426). Self-adherent silicone polymer microgrid arrays (50 × 50 μm; Microsurfaces, cat. #MGA-050-02) were affixed to the bottom of each well to enable separate observation of individual lytic events.

Effector cells were stained with CellVue Claret Far Red dye (Sigma Aldrich, cat. #12352207) according to the manufacturer’s instructions prior to coincubation. MOLM-14 CD117^High^ cells were used as targets at an E:T ratio of 1:1, with 4 × 10^4^ cells per well. CD117xCD3 TCE was added at a final concentration of 500 ng/mL, whereas CD33xCD28 IgG4-scFv_2_ or CD28-IgG4 was added at 0.5 nmol/L. For the controls, same volumes of PBS were added.

Time-lapse imaging was performed using a Nikon Ti2-E Eclipse microscope equipped with a linear-encoded motorized stage, Orca Flash 4.0 V2 camera (Hamamatsu), Spectra X fluorescent light source (Lumencor), a 10× CFI Plan Apochromat λ objective (NA 0.45), and an incubation chamber. Fluorescence detection was achieved using appropriate filter sets (AHF): CellVue Claret Far Red (620/60; 660LP; 700/75) and propidium iodide (550/32; 585LP; 605/15). Brightfield images were acquired using white light from the Spectra X and a custom motorized mirror controlled via Arduino UNO Rev3 (Arduino). Typically, 16 positions per microgrid were imaged at 10-minute intervals for brightfield and at 10- or 30-minute intervals for propidium iodide and CellVue channels over a period of up to 72 hours.

Single-cell tracking was performed using custom-written software (39–41). For additional *in vitro* experiments, brightfield images were acquired using an upright epifluorescence microscope (DM5500-B, Leica) at 5× magnification (HC PL FLUOTAR 5x/0.15 Leica objective). Leica LAS-X software was used for microscope control, image acquisition, and analysis.

### Statistical analysis

Unless otherwise specified, all data are presented as the mean ± standard error of the mean (SEM). Statistical analyses were performed using GraphPad Prism (version 10, RRID: SCR_002798), applying the appropriate statistical models as indicated in each figure legend. Technical replicates are plotted individually in figures; however, values were averaged within each experiment, and all statistical tests were conducted using the biological replicates. A *P* value of <0.05 was considered statistically significant. The following notation was used to indicate levels of significance: *, *P* < 0.05; **, *P* < 0.01; ***, *P* < 0.001; ****, *P* < 0.0001.

## Results

### Engineering of a CD33xCD28 IgG4-scFv_2_ bispecific antibody for dual targeting of T cells and AML antigens

**Figure 1.**
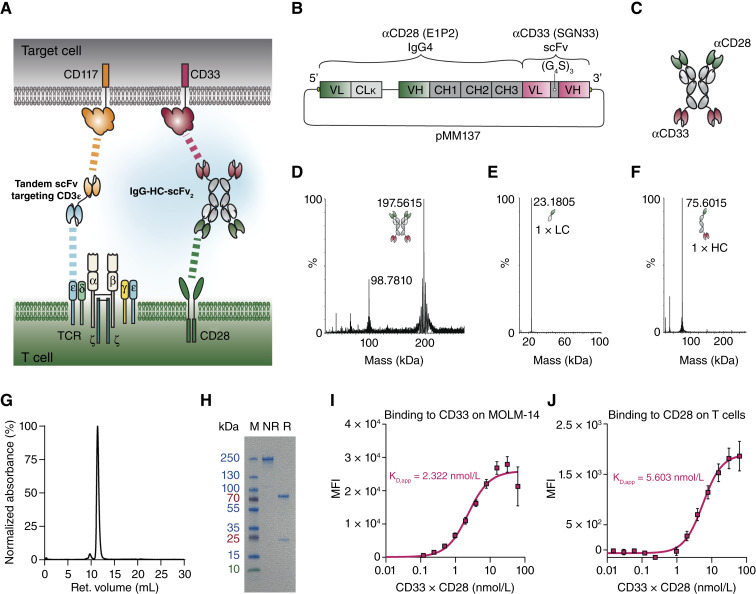
Characterization of CD33xCD28 IgG4-scFv_2_ TCE. **A,** Illustration of a combination of a bispecific TCE targeting TAA1 (CD117) on tumor cells and CD3ε on T cells with a bispecific TCE targeting TAA2 (CD33) on tumor cells and CD28 on T-cells. **B,** Plasmid map of the CD33xCD28 IgG4-scFv_2_ construct. **C,** Protein structure of the CD33xCD28 IgG_4_-scFv_2_ construct. **D–F,** CD33xCD28 IgG4-scFv_2_ analysis by mass spectrometry in nonreduced (**D**) and reduced (**E** and **F**) conditions. **G,** Size-exclusion chromatography of CD33xCD28 IgG4-scFv_2_. **H,** SDS-page analysis of CD33xCD28 IgG4-scFv_2_ under nonreducing (NR) and reducing (R) conditions M = protein ladder indicating the molecular size (kDa). **I,** Binding of CD33xCD28 IgG4-scFv_2_ to CD33 on MOLM-14 CD117^High^GFP^+^Luc^+^ cells. **J,** Binding of CD33xCD28 IgG4-scFv_2_ to CD28 on peripheral blood T cells. Binding capacity was assessed by the titration of the bispecific antibody and detected by anti-human IgG antibody. MFI was normalized to background fluorescence. Apparent K_D_ was calculated by nonlinear regression. Mean ± SD from three independent experiments, each plated in duplicates.

Figure 1A illustrates the conceptual framework of this study, in which an already described bispecific antibody construct, directed against CD3 on T cells and CD117 on AML cells, is combined with a novel bispecific antibody construct, targeting CD28 on T cells and CD33 on AML cells (29). The CD28 bispecific antibody was designed in an IgG-HC-scFv format, consisting of an anti-CD28 IgG4 backbone (clone E1P2, patent WO2024/018069; ref. 36), kindly provided by Philochem/Philogen, and the SGN33 moiety for CD33 targeting (37), incorporated as a scFv fused to the C-terminal end via a short GS linker. Figure 1B and C depict the schematic design of the expression vector and the resulting protein structure, respectively. The full amino acid sequence is reported in Supplementary Fig. S1. Figure 1D shows the LC-MS of the nonreduced protein, which seems close to the expected molecular weight of 194,664 Da (the lower peak at 98.781 is a harmonic deconvolution artifact). Upon reduction, the light chain (expected 23,184 Da) and heavy chain (expected 74,165 Da) are visible in Fig. 1E and F, respectively. The heavy chain shows an observed mass slightly above its expected size, likely due to posttranslational modifications, such as glycosylation (42). Size-exclusion chromatography (Fig. 1G) confirmed that more than 90% of the protein exists in a monomeric form. SDS-PAGE analysis further validated the LC-MS findings for the reduced sample, which appeared at approximately the expected molecular weight, whereas the unreduced protein ran slightly higher (250 kDa instead of 200 kDa), possibly due to migration problems (Fig. 1H). Figure 1I shows binding to CD33 on MOLM-14 CD117^High^GFP^+^Luc^+^ cells with an apparent dissociation constant (K_D_,_app_) of 2.322 nmol/L, whereas Fig. 1J demonstrates binding to CD28 on T cells with a K_D,app_ of 5.603 nmol/L.

### CD33xCD28 IgG4-scFv_2_ requires CD3-mediated signal 1 for T-cell activation

**Figure 2.**
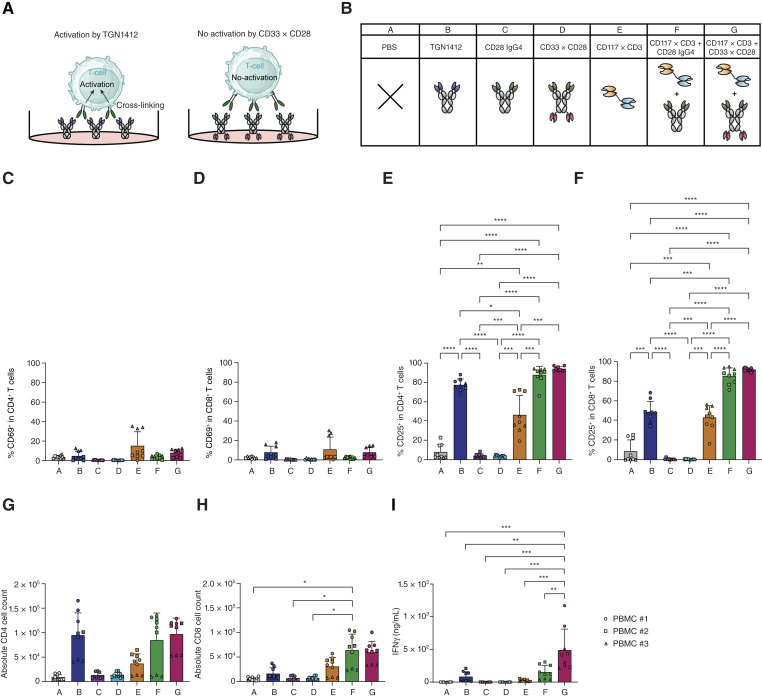
CD33xCD28 IgG4-scFv_2_ TCE does not activate T cells in the absence of TCR stimulation. **A,** Schematic representation of superagonism observed with the CD28 TGN1412, in which membrane-proximal binding and immobilization facilitate cross-linking of CD28 receptors leading to T-cell activation in the absence of TCR engagement (left) and of agonism observed with CD33xCD28 IgG4-scFv_2_ with no T-cell activation (right). **B,** Graphical legend indicating the different molecules added in the assay in **C–I**. Plates were initially coated with the respective antibody constructs at a concentration of 10 μg/mL, followed by incubation with PBMCs. After 96 hours, the different conditions were analyzed by flow cytometry (**C–H**) or ELISA (**I**). Data are represented as the mean ± SD from three healthy donor–derived PBMCs, each plated in duplicates. Each donor PBMC is represented with a different symbol. **C** and **D,** Percentage of CD69^+^ in CD4^+^ T cells (**C**) and in CD8^+^ T cells (**D**). **E** and **F,** Percentage of CD25^+^ in CD4^+^ T cells (**E**) and in CD8^+^ T cells (**F**). **G** and **H,** Absolute CD4^+^ T-cell (**G**) and CD8^+^ T-cell counts (**H**). **I,** IFNγ quantification in the supernatant of the different conditions. Statistical analysis for all the graphs was conducted using one-way ANOVA; *, *P* < 0.05; **, *P* < 0.01; ***, *P* < 0.001; ****, *P* < 0.0001. [**A,** Created in BioRender. Caiado, F. (2026) https://BioRender.com/5d04w2s.]

We next evaluated whether CD33xCD28 IgG4-scFv_2_ would lead to T-cell activation in the absence of signal 1, i.e., in the absence of TCR/CD3 stimulation. To benchmark superagonistic CD28 activity, we included TGN1412 as a positive control. TGN1412 is a well-characterized CD28 superagonist that, in a first-in-human trial, induced severe cytokine release syndrome due to potent immunostimulatory activity in the absence of TCR engagement (43, 44). This activation was driven by cross-linking of CD28 receptors on T cells, which might have been facilitated *in vivo* by Fcγ receptor–bearing accessory cells, immobilizing the antibody via its Fc region (45, 46). Importantly, TGN1412 binds a membrane-proximal epitope on CD28, which further enhances its ability to induce receptor clustering and activation. Figure 2A depicts the experimental setup, in which TGN1412 would activate T cells (positive control, left) and the respective activity of CD33xCD28 IgG4-scFv_2_ is evaluated (test situation, right), ideally not leading to T-cell activation. We further modified the test setup by adding indicated antibody constructs in Fig. 2B (TGN1412 as positive control, CD28 IgG4, CD33xCD28 IgG4-scFv_2_, CD117xCD3 TCE, or combinations of anti-CD28 with CD117xCD3).

We first assessed CD28 expression on CD4^+^ and CD8^+^ T cells. As shown in Supplementary Fig. S2A, CD28 was uniformly expressed on CD4^+^ T cells, whereas about 68% to 78% of CD8^+^ T cells expressed it.

After 96 hours of T-cell incubation, we evaluated expression of CD69 and CD25 as well as absolute counts of CD4 and CD8 expressing T cells (Fig. 2C–H). CD4 and CD8 T cells were highly activated as determined by CD25 expression in the presence of TGN1412, CD117xCD3, CD117xCD3, and CD28 IgG4, as well as CD117xCD3 and CD33xCD28 IgG4-scFv_2_ (Fig. 2E and F). Also, CD4 cell counts increased significantly under the same conditions (Fig. 2G), although this was true to a lesser extent for CD8 T cells (Fig. 2H). Importantly, PBS, CD28 IgG4, and CD33xCD28 IgG4-scFv_2_ did not lead to T-cell activation and proliferation. Moreover, significant IFNγ increase in the supernatant was only observed in the setting of CD33xCD28 IgG4-scFv_2_ in combination with CD117xCD3 (Fig. 2I).

In sum, these data demonstrate that CD33xCD28 IgG4-scFv_2_ does not induce T-cell stimulation in the absence of signal 1, i.e., TCR/CD3 stimulation, at least in this highly controlled *in vitro* setting.

### CD33xCD28 IgG4-scFv_2_ enhances CD117xCD3-induced AML target cell line lysis by T cells

We next evaluated the combination of CD33xCD28 IgG4-scFv_2_ with CD117xCD3 TCE, focusing on T-cell activation, proliferation, cytokine release, and overall cytotoxicity against the AML cell lines MOLM-14 and HL-60, transduced to express varying levels of CD117 as well as against endogenously CD117-expressing KASUMI-1 cells.

**Figure 3.**
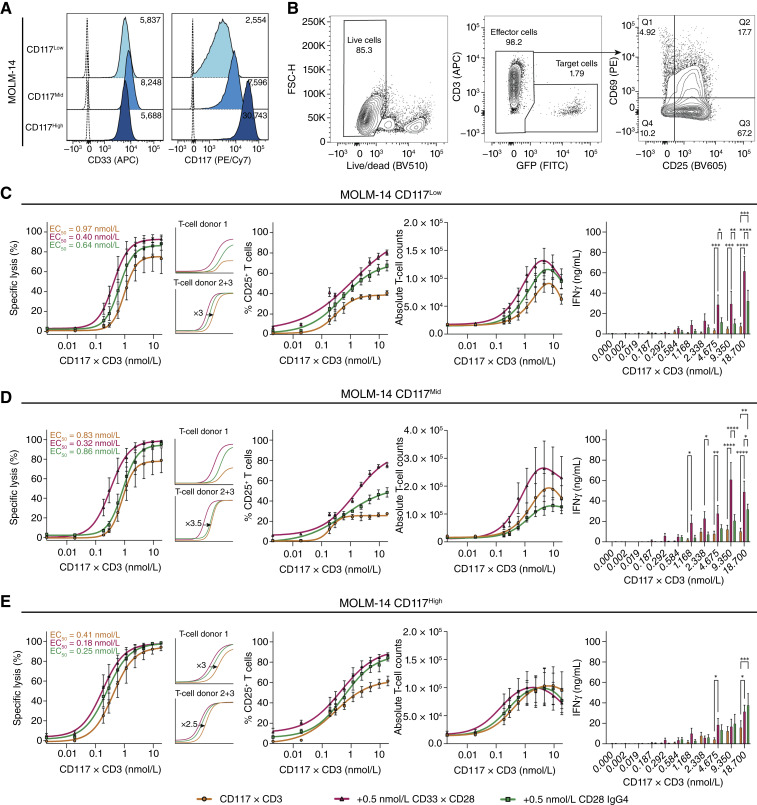
The addition of CD33xCD28 IgG4-scFv_2_ to CD117xCD3 TCE increases the cytotoxicity of T cells against human AML cell lines with various degrees of CD117 expression. **A,** Representative histograms of CD117 and CD33 expression on MOLM-14 AML cells, transduced to express CD117 at low, intermediate, and high levels in comparison with unstained controls of each cell line (dotted line). **B,** Example of flow cytometry and respective gating strategy at 96 hours of coculture of MOLM-14 cells with healthy donor–derived T cells at E:T = 1:1 and CD117xCD3 with 0.5 nmol/L of binder molecules (specific sample: MOLM-14 CD117^Low^GFP^+^Luc^+^ with 1.168 nmol/L CD117xCD3 and 0.5 nmol/L CD33xCD28 IgG4-scFv_2_). **C–E,** MOLM-14 CD117 low (**C**), mid (**D**), or high (**E**) were cocultured with healthy donor–derived T cells at E:T = 1:1 and indicated amounts of CD117xCD3, whereas constant amounts of 0.5 nmol/L of either CD33xCD28 IgG4-scFv_2_ or anti-CD28 antibodies were added. Analysis was performed via flow cytometry at 96 hours for specific lysis (first column), CD25^+^ expression on T cells (second column), absolute T-cell counts (third column), and ELISA for IFNγ (fourth column). Mean ± SEM from three healthy donor–derived T cells, each analyzed in duplicates. Statistical analysis for all the graphs was conducted using two-way ANOVA; *, *P* < 0.05; **, *P* < 0.01; ***, *P* < 0.001; ****, *P* < 0.0001.

Figure 3A shows the natural expression of CD33 (the first TAA target) and the induced levels of CD117 (the second TAA target). AML blasts typically lack CD80 and show low, heterogeneous expression of CD86, the natural ligands of CD28, leading to insufficient costimulation and immune evasion in AML (47, 48). CD80 was not or only minimally expressed, whereas CD86 was expressed at very low levels on MOLM-14, HL-60, and KASUMI-1 (Supplementary Fig. S2B–S2D). This supports the rationale for using a CD28 agonist in order to bypass the need for endogenous costimulatory ligands.

The three MOLM-14 variants were cocultured with unexpanded healthy donor–derived T cells at a 1:1 E:T ratio for 96 hours. Cultures were treated with varying concentrations of CD117xCD3 TCE and either CD33xCD28 IgG4-scFv_2_ or CD28 IgG4 at four different doses. Figure 3B shows a representative gating strategy used to analyze flow cytometry data. Live cells were gated first, followed by the separation of effector and target cells using CD3 and GFP. Effector cells were then assessed for CD69 and CD25 expression to evaluate activation.

Figure 3C–E present results using 0.5 nmol/L of either CD33xCD28 IgG4-scFv_2_ or CD28 IgG4. Across all three MOLM-14 lines, no cytotoxicity was observed when small amounts of CD117xCD3 TCE (i.e., 0.0019 nmol/L) were used alone or when combined with CD33xCD28 IgG4-scFv_2_ or CD28 IgG4. Cytotoxicity increased in a dose-dependent manner with CD117xCD3 TCE and correlated with CD117 expression, in which higher CD117 levels yielded stronger monotherapy responses. Importantly, combining CD117xCD3 TCE with CD33xCD28 IgG4-scFv_2_ led to a 2- to 3-fold reduction in EC_50_ value across all MOLM-14 variants. Notably, a beneficial effect was also observed in the MOLM-14 CD117^Low^ GFP^+^Luc^+^ cell line, which rather reflects CD117 expression levels typically observed in AML patients, as demonstrated previously (25). In addition, CD33xCD28 IgG4-scFv_2_ outperformed CD28 IgG4 in boosting cytotoxicity, and when analyzing individual donor responses, the combination treatment revealed even more apparent effects [Fig. 3C–E (left)]. T-cell activation (CD69 and CD25), proliferation, and IFNγ release positively correlated with cytotoxicity [Fig. 3C–E (second to fourth from the left)].

To explore dose dependency, we tested lower and higher concentrations of CD33xCD28 IgG4-scFv_2_ (Supplementary Fig. S3A–S3F). At suboptimal doses, no significant improvement over monotherapy was observed. At high doses of 10 nmol/L, the benefit seen at 0.5 nmol/L was diminished, likely due to target saturation, which impairs the ability of a single bispecific molecule to bridge effector and target cells, a phenomenon previously described (29, 49, 50). This effect was not observed with CD28 IgG4, in which higher doses failed to provide additional benefit.

To validate these findings, we repeated the experiments using HL-60 and KASUMI-1 AML cell lines (Supplementary Figs. S4A, S4B, S5A, and S5B). The results mirrored those from MOLM-14, with EC_50_ reductions ranging from 3.6- to 10.5-fold for HL-60 and 6.4-fold for KASUMI-1 [Supplementary Figs. S4C–S4E, and S5C (left)]. With the KASUMI-1 cells, we observed slightly elevated cytotoxicity at very low concentrations of CD117xCD3 TCE when 0.5 nmol/L CD33xCD28 IgG4-scFv_2_ was present, possibly due to alloreactivity. As with MOLM-14 cells, specific lysis positively correlated with T-cell activation and proliferation [Supplementary Figs. S4C–S4E, and S5C (second and third)].

Together, these results demonstrate that CD33xCD28 IgG4-scFv_2_ significantly enhances CD117xCD3-induced lysis of CD117^+^CD33^+^ AML target cell lines by T cells, even at lower levels of CD117 expression that resemble those detected on AML patient blasts (29).

### Sustained T-cell activity against AML cells upon repeated target-cell exposure

The use of T-cell engagers has been associated with T-cell exhaustion due to repeated stimulation by target cells. To assess this in part, we performed short-term rechallenging experiments as shown in Supplementary Fig. S6A. MOLM-14 CD117^High^ cells were cocultured at an E:T ratio of 1:1 with healthy donor–derived T cells in 96-well plates. Cultures were either left untreated, treated with 9.35 nmol/L CD117xCD3 TCE alone, or treated with the combination of CD117xCD3 TCE and 0.5 nmol/L CD33xCD28 IgG4-scFv_2_ for 3 days.

After this initial incubation, cells were analyzed by flow cytometry to determine MOLM-14 CD117^High^ and T-cell counts. T cells were then rechallenged by adding fresh MOLM-14 CD117^High^ cells to reestablish an E:T ratio of 1:1, together with fresh antibody. Cytotoxicity was assessed again after an additional 3 days (Supplementary Fig. S6A).

MOLM-14 CD117^High^ cells were eliminated in both monotherapy- and combination-treatment conditions at day 3 and remained absent at day 6 following rechallenge, i.e., reexposure to target cells, indicating that T cells retained the capacity to robustly eliminate tumor cells upon a second encounter (Supplementary Fig. S6B). T cells expanded substantially, particularly by day 6, regardless of rechallenge status, with the greatest expansion observed in the combination-treatment group (Supplementary Fig. S6C).

CD25 expression and IFNγ release were highest in conditions in which T cells were actively eliminating MOLM-14 CD117^High^ cells (i.e., day 3 and day 6 rechallenge settings; Supplementary Fig. S6D and S6E). Supplementary Figure S6F shows the proportion of CD4^+^ and CD8^+^ T cells, with a slight increase in CD8^+^ T cells at day 6 in treatment conditions, independent of rechallenge.

Finally, expression of the exhaustion markers TIM-3 and LAG-3 was highest at day 3 in treatment samples but decreased by day 6 following rechallenge and decreased even further by day 6 in the no-rechallenge condition (Supplementary Fig. S6G).

Together, these results demonstrate that in a short-term rechallenge setting, T cells remain functional and capable of eliminating and controlling AML cells upon a second stimulation.

### CD33xCD28 IgG4-scFv_2_ leads to faster and more specific CD117xCD3-induced AML target cell line lysis by T cells

**Figure 4.**
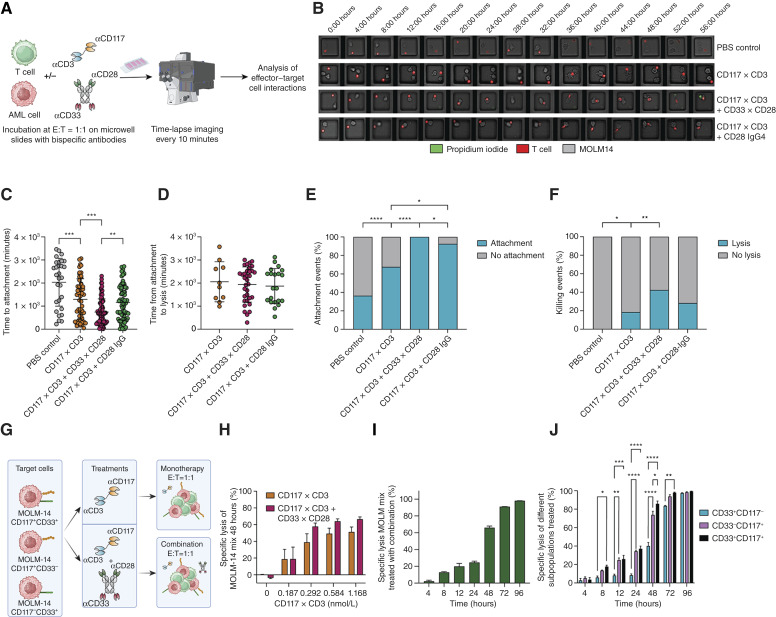
Combination of CD117xCD3 TCE and CD33xCD28 IgG4-scFv_2_ mediates faster and more selective T-cell lysis of MOLM-14 target cells. **A,** Schematic of the experimental workflow of time‐lapse imaging process to study effector–target cell interactions over time. Cells were cocultured at an E:T ratio of 1:1 for up to 72 hours on microgrids which are glued to chamber slides to allow for quantification of single attachment and lysis events. After the required criteria are met for imaging (i.e., good movie quality allowing for cell traceability and high cell viability of the cells over the course of imaging), individual time‐lapse movies were acquired in those wells in which an E:T ratio of 3:1 to 1:3 was achieved. **B,** Representative time-lapse images of MOLM-14 CD117^high^ cells (gray) and T cells (red), incubated either without binders, with 500 ng/mL CD117xCD3, with CD117xCD3 in combination with CD33xCD28 IgG4-scFv_2_ (0.5 nmol/L), or with CD117xCD3 and CD28 IgG4 (0.5 nmol/L), are shown. Target cell lysis is marked by PI influx (green). **C** and **D,** Time to target cell attachment and time from target cell attachment to lysis, i.e., PI influx between different conditions. T cells with CD117xCD3 in combination with CD33xCD28 IgG4-scFv_2_ showed fastest target cell attachment, whereas time from attachment to killing was statistically different between conditions (all results from two healthy donors, statistical analysis conducted using one-way ANOVA or unpaired Student *t* test; *, *P* < 0.05; **, *P* < 0.01; ***, *P* < 0.001; ****, *P* < 0.0001). **E,** A total of 240 individual time-lapse imaging movies were analyzed for quantification of effector–target cell interactions (*n* = 80, 80, 80, and 80 for each group). Quantification of target-cell interaction showed complete T-cell attachment to target cells with CD117xCD3 in combination with CD33xCD28 IgG4-scFv_2_. **F,** Following attachment, target cells in individual wells were tracked for T cell–induced lysis. Maximum lysis was observed with the combination of CD117xCD3 and CD33xCD28 IgG4-scFv_2_ (right; *n* analyzed = 29, 54, 80, 74 for each group). All results are from two healthy donors. Statistical analysis was conducted using *χ*^2^ test; *, *P* < 0.05; **, *P* < 0.01; ***, *P* < 0.001; ****, *P* < 0.0001. **G,** Representation of the experimental setup of the specificity assay generated with BioRender.com. An equal mixture of MOLM-14 cells (either CD117^+^CD33^+^, CD117^+^CD33^−^, or CD117^−^CD33^+^) were coincubated with unexpanded healthy donor–derived T cells at an E:T ratio of 1:1 and either CD117xCD3 alone or in combination with 0.5 nmol/L CD33xCD28 IgG4-scFv_2_. Cocultures were evaluated by flow cytometry at 4, 8, 12, 24, 48, 72, and 96 hours. All experiments executed with T cells from three healthy donors, each analyzed in duplicates. **H,** Percentage specific lysis of MOLM-14 mix treated with either CD117xCD3 alone at several concentrations or in combination with 0.5 nmol/L CD33xCD28 IgG4-scFv_2_. **I,** Percentage specific lysis of mixed MOLM-14 cells and individual MOLM-14 subpopulations (**J**), incubated with T cells and the combination of 1.168 nmol/L CD117xCD3 and 0.5 nmol/L CD33xCD28 IgG4-scFv_2_ at indicated time points. Statistical analysis was conducted using two-way ANOVA; *, *P* < 0.05; **, *P* < 0.01; ***, *P* < 0.001; ****, *P* < 0.0001. [**A,** Created in BioRender. Caiado, F. (2026) https://BioRender.com/sbcghjr; **G,** Created in BioRender. Caiado, F. (2026) https://BioRender.com/1gojldm.]

Having demonstrated that CD33xCD28 IgG4-scFv_2_ significantly enhances the efficacy of CD117xCD3 TCE, we next investigated the dynamics of effector–target cell interactions and lysis. To this end, we performed time-lapse imaging of MOLM-14 CD117^High^GFP^+^Luc^+^ cells, cocultured with healthy donor–derived T cells, as previously described (29). Cultures were supplemented with CD117xCD3 TCE or with CD117xCD3 TCE in combination with 0.5 nmol/L of either CD33xCD28 IgG4-scFv_2_ or CD28 IgG4 (Fig. 4A). For analysis, a range of E:T ratios from 1:3 to 3:1 was considered. Figure 4B illustrates the successful detection of cell interactions and killing events over time across various conditions. A total of 80 individual wells per condition were analyzed for attachment and lysis. As shown in Fig. 4C, cell attachment occurred even in the PBS control but with delayed kinetics compared with the treatment groups. The fastest time to attachment was observed in the combination of CD117xCD3 TCE with CD33xCD28 IgG4-scFv_2_, i.e., in a situation in which two bispecifics bridge AML target cells and T cells. In contrast, slightly slower attachment times were measured when only CD117xCD3 was used, independent of the addition of T-cell binding CD28-IgG4. Interestingly, the time from attachment to lysis did not differ significantly between conditions (Fig. 4D). However, nearly all close-contact events lead to stable attachment in the CD117xCD3 TCE with CD33xCD28 IgG4-scFv_2_ group (Fig. 4E). Notably, the addition of the CD28 IgG4 molecule to the CD117xCD3 TCE also increased the proportion of close-contact events that converted into stable attachments even in the absence of CD33. IgG4 antibodies retain the ability to bind FcγRI, FcγRIIA, and FcγRIIB when Fc-silencing mutations are not introduced (51). In our construct, no Fc-silencing mutations were incorporated, and MOLM-14 cells are known to express Fcγ receptors (52). This provides a plausible explanation for why the CD28 IgG4 construct enhances cell–cell attachment independently of the presence of CD33 scFv. Among these attached cells, the highest proportion of lysis events was again observed in the combination treatment, underscoring the enhanced efficiency of dual bispecific targeting (Fig. 4F). These results suggest that CD33xCD28 IgG4-scFv_2_ not only accelerates initial T-cell engagement but also improves the effectiveness of cell-mediated killing.

To assess whether dual targeting also improves lysis specificity, we cocultured healthy donor–derived T cells with a mix of MOLM-14 cells at a 1:1 E:T ratio (Fig. 4G). The mix consisted of equal amounts of CD117^+^CD33^+^, CD117^+^CD33^−^, and CD117^−^CD33^+^ MOLM-14 cells. Cultures were treated with CD117xCD3 TCE or with CD117xCD3 TCE in combination with CD33xCD28 IgG4-scFv_2_. Target cell lysis was evaluated over the course of 96 hours. A representative plot at 48 hours (Fig. 4H) shows that the combination therapy slightly enhanced lysis compared with CD117xCD3 TCE monotherapy in the combined target cell mix, despite the presence of subpopulations lacking one of the target antigens. When analyzing cytotoxicity over time in cultures treated with 1.168 nmol/L CD117xCD3 TCE and 0.5 nmol/L CD33xCD28 IgG4-scFv_2_, we observed progressive lysis, approaching near-complete cytotoxicity by 72 hours, even in cells lacking CD117 (Fig. 4I). This is likely due to bystander killing effects in a single well, as previously described (25). Dissecting the MOLM-14 mix into its subpopulations revealed that between 8 to 24 hours, CD117^−^CD33^+^ cells were minimally lysed, consistent with the absence of signal 1 delivery via CD117xCD3 TCE. At 24 hours, a trend emerged, showing preferential lysis of CD117^+^CD33^+^ cells (engaging both antibodies) over CD117^+^CD33^−^ cells (engaging only CD117xCD3 TCE). This difference became statistically significant at 48 hours (Fig. 4J).

In summary, these results demonstrate that combining CD33xCD28 IgG4-scFv_2_ with CD117xCD3 TCE not only enhances overall cytotoxicity but also improves antigen-specific targeting, preferentially eliminating target cells coexpressing CD33 and CD117, thereby increasing therapeutic precision in heterogeneous disease settings such as AML.

### CD33xCD28 IgG4-scFv_2_ enhances CD117xCD3-induced primary AML cell lysis by T cells

AML is a highly heterogeneous disease, characterized by variable expression of surface markers such as CD33 and CD117. This heterogeneity presents a complex and clinically relevant challenge. To assess the therapeutic potential of CD33xCD28 IgG4-scFv_2_ in this context, we evaluated its activity against primary AML samples in combination with CD117xCD3 TCE.

**Figure 5.**
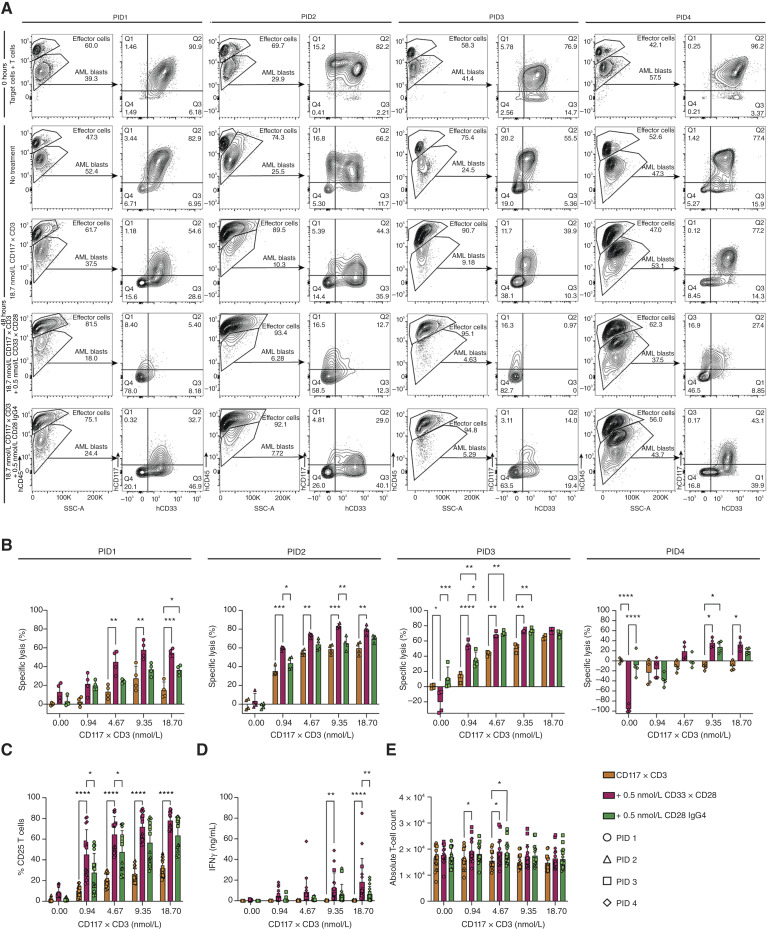
CD33xCD28 IgG4-scFv_2_ in combination with CD117xCD3 TCE mediates more effective lysis of primary AML cells. **A,** Representative flow cytometry plots showing CD117 and CD33 expression on four different primary human AML blast populations (CD45dim) upon coculture with healthy donor–derived T cells at an E:T ratio of approximately 1:1. Cells were incubated with antibody constructs as indicated (CD117xCD3 at the indicated concentrations, in combination with 0.5 nmol/L CD33xCD28 IgG4-scFv_2_ or 0.5 nmol/L CD28 IgG4). Plots are shown for 0 and 48 hours. **B,** Percentage of specific lysis of CD45^dim^CD3^−^ AML blasts of the individual patient samples after 48 hours. **C,** Combined percentage of CD25^+^ T cells. **D,** Combined IFNγ in supernatants of coculture. **E,** Combined proliferation of T cells. Data represent the mean ± SEM from two independent healthy donor–derived T-cell samples, each plated in duplicate. Statistical significance was determined using two-way ANOVA; *, *P* < 0.05; **, *P* < 0.01; ***, *P* < 0.001; ****, *P* < 0.0001.

Primary AML PBMCs were cocultured with healthy donor T cells at an approximate E:T ratio of 1:1 for 48 hours. Cultures were treated with varying concentrations of CD117xCD3 TCE in combination with either 0.5 nmol/L CD33xCD28 IgG4-scFv_2_ or CD28 IgG4. Figure 5A shows representative flow cytometry plots from four primary AML samples, captured at baseline and after 48 hours of coculture. At time point 0, all samples exhibited partial expression of CD117 and CD33 at varying levels. After 48 hours, untreated AML cells remained largely unaffected. Treatment with CD117xCD3 TCE alone led to a reduction in CD117^+^ cell populations. Notably, the combination of CD117xCD3 TCE with CD33xCD28 IgG4-scFv_2_ resulted in a more pronounced depletion of CD117^+^ cells, whereas the combination with CD28 IgG4 produced intermediate effects. Figure 5B depicts the individual cytotoxicity across four primary AML samples, with distinct symbols representing individual patients. The addition of CD33xCD28 IgG4-scFv_2_ to CD117xCD3 TCE enhanced the specific lysis in all four primary AML samples by an increase of up to 40%, compared with 22% when CD117xCD3 was combined with CD28 IgG4. This increased cytotoxicity correlated with elevated T-cell activation (Fig. 5C) and IFNγ release (Fig. 5D), whereas T-cell proliferation remained unchanged at the 48-hour time point (Fig. 5E). PID1 showed the highest benefit to the combination therapy, followed by PID2 and PID3, and lastly PID4. Furthermore, PID1 showed about 18% of lysis in the presence of only CD33xCD28 IgG4-scFv_2_, likely due to alloreactivity (Fig. 5B). The experiment was also performed at 96 hours for PID1 and PID2, in which, compared with 48 hours, PID1 showed more pronounced specific lysis in absence of CD117xCD3 TCE, T-cell activation persisted, and the combination produced substantially greater T-cell proliferation, resulting in more than a twofold increase in total T-cell numbers (Supplementary Fig. S7A–S7C).

In conclusion, these data demonstrate that CD33xCD28 IgG4-scFv_2_ significantly augments the cytotoxic activity of CD117xCD3 TCE in combination with T cells in a setting with antigen-heterogeneous primary human AML cells.

## Discussion

With enhanced bioengineering capacities, two new immunotherapeutic modalities, CAR T cells and TCEs, have been developed. Both direct T cells in an MHC/TCR-independent fashion toward target antigens, leading to target cell lysis. CAR T cells are T cells genetically modified to express a CAR, which, upon target antigen binding, transmits an activating signal via CD3ζ, mimicking TCR stimulation. For optimal CAR T-cell function, this signal is enhanced by in sequence engineered costimulatory molecules (CD28 or 41BB). Only these so-called second-generation CAR T cells, delivering both signals 1 and 2, have been proven effective in clinical settings (15, 49, 50, 53, 54). Advantages of CAR T cells are their high efficacy and their potential therapeutic durability as a “living drug”. Disadvantages are their complex, individual per-patient generation, their relatively low numbers compared with the patient overall T-cell compartment, and the difficulty of guiding their activity and controlling potential side-effects, once applied to the patient (55–58). TCEs, in contrast to CAR T-cells, are off-the-shelf CD3ε and target antigen–binding bispecific molecules. Advantages of TCEs are their immediate availability upon need, their theoretical capacity to activate the whole available T-cell compartment in a patient, thus increasing the E:T ratio, and their activity is controllable based on on-target half-lives (59). Disadvantages of TCEs are that they need to be repetitively applied and that they are overall less efficient compared with CAR T cells, possibly in part due to lack of T-cell costimulation (19, 59–61).

Currently, the best clinical results with both CAR T cells and, to a lesser extent, with TCEs are achieved by targeting non–MHC-restricted cell-of-origin surface antigens, such as CD19 and CD20 on B-cell neoplasia and BCMA and GPRC5D on plasma cell neoplasia (60, 62, 63). This leads to both malignant cell and healthy counterpart cell depletion, requiring, in many cases, temporary immunoglobulin substitution (64). To enhance the efficacy of CD3-directed TCEs, coapplication of agonistic CD28 engagers is attractive. However, antibody-based engagement of CD28 has progressed cautiously, largely due to massive cytokine release induced by a super-agonistic CD28 IgG4 (TGN1412) in healthy volunteers (65, 66). Most recent data, though, indicate the safety and efficacy of next-generation agonistic CD28 bispecific antibodies in preclinical and first clinical settings (20, 21, 67–70).

Immunotherapeutic approaches in AML and high-risk MDS are lagging behind the successes achieved in B-cell and plasma cell malignancies. AML cells express very few tumor-private antigens on MHC, and targeting cell-of-origin antigens leads to depletion of healthy HSPCs and myeloid cells (64). Thus, cell-of-origin targeting could serve as an alternative to the current nonspecific chemotherapy used prior to HSPC transplantation, although this approach would require the controlled termination of effector cell activity (64, 71, 72). We and others have nevertheless followed this concept by targeting CD117, expressed on healthy HSPC and AML cells, with blocking antibodies, antibody–drug conjugates, CAR T cells, adapter CAR T cells, and CD117xCD3 TCEs (25, 29, 34, 73, 74). We hypothesized that adding a second TAA (TAA2)-directed, bispecific agonistic CD28 costimulatory construct to the TAA1-directed CD117xCD3 TCE might have two beneficial effects: (i) enhancement of TCE-mediated lytic activity by promoting stronger T-cell expansion via IL2/CD25 signaling, upregulating survival pathways such as BcL-xL, driving a more active/memory-like T-cell phenotype, and increasing the recruitment of CD28 molecules within the artificial immunologic synapse (21, 67, 70, 75) and (ii) increased selectivity to double-antigen–expressing cells (25, 32, 76). This logic would require that the TAA1-directed TCE is leading lytic activity and thus should be as tumor-restricted as possible, whereas the TAA2-directed costimulatory activity could be directed to a more broadly expressed antigen, as this molecule should not activate T cells in the absence of signal 1, mediated by the CD117xCD3 TCE. This further implies that the duration of lytic activity should be determined by the half-life of CD117xCD3 TCE, allowing for longer half-life constructs for the TAA2-restricted CD28-stimulating molecule. Based on these considerations, we chose CD33, a myelopoiesis-restricted antigen, expressed on most AML, as TAA2 (25, 29, 32, 77).

Building on this concept, we here engineered and characterized CD33xCD28 IgG4-scFv_2_, a bispecific antibody. Our results demonstrate that CD33xCD28 IgG4-scFv_2_ is a stable molecule that binds its targets with high affinity and, importantly, similar as the parental anti-CD28 IgG antibody, does not induce T-cell activation in the absence of CD3-mediated signal 1, addressing the key safety concern associated with CD28 superagonism. *In vitro*, the addition of CD33xCD28 IgG4-scFv_2_ to CD117xCD3 TCE improved T-cell activation, proliferation, and cytokine release, resulting in enhanced cytotoxicity. Notably, this effect was also observed under low CD117 target-expression conditions, which reflect the antigen levels typically found in patients with AML (29). The observed stronger T-cell activation in the combination of CD117xCD3 TCE with CD33xCD28 IgG4-scFv_2_ versus weaker T-cell activation in the combination of CD117xCD3 TCE with anti-CD28 IgG4 might be due to better integration of the costimulatory signal in the formed immune synapse. Alternatively, the dual-binding of CD33xCD28 IgG4-scFv_2_ might enhance the signal delivered by CD117xCD3 TCE via CD3 stimulation through stronger T-cell binding and prolonged productive synapse formation, a possibility not tested in our experiments (78–80).

Continuous antigen exposure and overstimulation have been shown to impair T-cell fitness through exhaustion and/or activation-induced cell death, reducing therapeutic efficacy (81–83). We sought to address this using a short-term rechallenge experiment, which demonstrated that upon reexposure to AML cells, T cells retained their lytic capacity and were able to eliminate tumor cells repetitively.

Time-lapse imaging revealed that dual-targeting accelerates T-cell engagement and increases the proportion of killing events. Although the CD28 IgG4 construct alone showed some improvement in T-cell engagement, potentially via Fc–FcγR interactions, such Fc-mediated binding could represent a theoretical potential “off-target” mechanism for the CD33xCD28 IgG4-scFv_2_ molecule. This will need to be assessed more closely by comparing the relative affinities of Fc-FcγR versus CD33 binding, as the higher-affinity interaction is expected to dominate target engagement and thereby influence where the molecule preferentially localizes. Furthermore, in the CD33xCD28 IgG4-scFv_2_ format, FcγR-expressing cells may have reduced ability to engage the Fc region due to steric hindrance introduced by the CD33-binding scFv, which is attached to the IgG4 backbone via a very short “GS” linker. This configuration may restrict Fc accessibility. Future studies assessing the affinities of Fc–FcγR and CD33 binding, as well as evaluating the impact of steric constraints, will be important to determine the likelihood and extent of potential Fc-mediated off-target interactions. Moreover, the combination therapy preferentially eliminated CD117^+^CD33^+^ cells over single-antigen–expressing cells, reinforcing the value of multi-antigen targeting to improve specificity and efficacy.

Finally, CD33xCD28 IgG4-scFv_2_ combined with CD117xCD3 TCE was effective *in vitro* against primary AML samples. The activity of this combination requires sufficient CD117 expression, which was present in most cells within the samples tested. This also implies that not all AML subtypes may benefit equally, as more differentiated forms, such as monocytic AML, often express less CD117 and may therefore show reduced responsiveness. In our primary AML samples tested, a striking positive effect was observed in PID4, in which the addition of CD33-delivered CD28 costimulation was able to rescue the unresponsiveness to CD117xCD3 monotherapy.

However, our study does not address several important aspects and potential limitations. This includes *in vivo* application and scheduling of TCE as a single molecule and in combination (81–83). Additionally, engagement of the PD-1/PD-L1 axis can suppress CD28 signaling, negating its benefits (67, 69). These insights have prompted ongoing studies and clinical trials exploring combinations of CD28 agonists with immune checkpoint inhibitors (67, 69). Also, we here only focused on two AML and healthy HSPC-associated antigens, whereas other target antigens, potentially even AML-private, MHC-restricted TAA1 (the only TAA which could spare patients from subsequent HSPC transplantation) in combination with more broadly expressed TAA2 as, e.g., CD123, CD135, CLL-1 (CD371), or SICLEC‐6, remain to be explored (84).

Taken together, our findings highlight the promise of CD33xCD28 IgG4-scFv_2_ as a costimulatory enhancer in bispecific antibody therapy for AML. By improving both potency and specificity, this dual-targeting approach may offer a viable path forward in treating antigen-heterogeneous malignancies.

## Supplementary Material

Supplementary Figure S1 Figure S1 shows the amino acid sequence of CD33xCD28 IgG4-scFv2.

Supplementary Figure S2 Figure S2 shows the expression of CD28 on T-cells and CD80/Cd86 on MOLM-14, HL-60 and KASUMI-1 cell lines.

Supplementary Figure S3 Figure S3 shows the dose-dependent effects of CD33xCD28 IgG4-scFv2 on MOLM-14 cell lysis, induced by CD117xCD3 and T-cells.

Supplementary Figure S4 Figure S4 shows the dose-dependent effects of CD33xCD28 IgG4-scFv2 on HL-60 cell lysis, induced by CD117xCD3 and T-cells.

Supplementary Figure S5 Figure S5 shows the dose-dependent effects of CD33xCD28 IgG4-scFv2 on KASUMI-1 cell lysis, induced by CD117xCD3 and T-cells.

Supplementary Figure S6 Figure S6 shows that T-cells maintain specific lytic activity after repetitive challenge with MOLM-14 CD117High cells.

Supplementary Figure S7 Figure S7 shows the increased T-cell proliferation at 96h upon addition of CD33xCD28 IgG4-scFv2 to CD117xCD3 TCE in T-cell and primary AML cell co-culture.

Supplementary Table S1 Table S1 shows the list of antibodies used for flow cytometry.

Supplementary Table S2 Table S2 shows the list of primary AML patient samples and their genetic characteristics.

## Data Availability

The datasets generated and/or analyzed during the current study are available from the corresponding author upon request.
