# Supplementary material for: Enhancement of CD117-Targeted Bispecific T-cell Engagement by CD33-Targeted Bispecific T-cell Costimulation in Acute Myeloid Leukemia
Source: Cancer Res Commun. 2026 Apr 27;6(4):946–60. doi: 10.1158/2767-9764.CRC-25-0672 (PMC13114487; doi:10.1158/2767-9764.CRC-25-0672)
Supplement: Supplementary Figure S1 — Figure S1 shows the amino acid sequence of CD33xCD28 IgG4-scFv2. [file crc-25-0672_supplementary_figure_s1_suppsf1.pdf]

Supplementary Figure S1

Light chain:
DIQMTQSPSSLSASVGDRVTITCRASQSISSYLNWYQQKPGKAPKLLIYAASSLQSG
VPSRFSGSGSGTDFTLTISSLQPEDFATYYCQQGGMPPDTFGQGTKVEIKRTVAAP
SVFIFPPSDEQLKSGTASVVCLLNNFYPREAKVQWKVDNALQSGNSQESVTEQDS
KDSTYSLSSTLTLSKADYEKHKVYACEVTHQGLSSPVTKSFNRGEC

Heavy chain-scFv2:
EVQLLESGGGLVQPGGSLRLSCAASGFTFSSYAMSWVRQAPGKGLEWVSAISGS
GGSTYYADSVKGRFTISRDNSKNTLYLQMNSLRAEDTAVYYCAKRYIAFDYWGQG
TLVTVSSASTKGPSVFPLAPCSRSTSESTAALGCLVKDYFPEPVTVSWNSGALTSG
VHTFPAVLQSSGLYSLSSVVTVPSSSLGTKTYTCNVDHKPSNTKVDKRVESKYGPP
CPPCPAPEFLGGPSVFLFPPKPKDTLMISRTPEVTCVVVDVSQEDPEVQFNWYVD
GVEVHNAKTKPREEQFNSTYRVVSVLTVLHQDWLNGKEYKCKVSNKGLPSSIEKTI
SKAKGQPREPQVYTLPPSQEEMTKNQVSLTCLVKGFYPSDIAVEWESNGQPENNY
KTTPPVLDSDGSFFLYSRLTVDKSRWQEGNVFSCSVMHEALHNHYTQKSLSLSLG
GSDIQMTQSPSSLSASVGDRVTITCRASESVDNYGISFMNWFQQKPGKAPKLLIYA
ASNQGSGVPSRFSGSGSGTDFTLTISSLQPDDFATYYCQQSKEVPWTFGQGTKVE
IKGGGGSGGGGSGGGGSQVQLVQSGAEVKKPGSSVKVSCKASGYTFTDYNMHW
VRQAPGQGLEWIGYIYPYNGGTGYNQKFKSKATITADESTNTAYMELSSLRSEDTA
VYYCARGRPAMDYWGQGTLVTVSS

Supplementary Figure S1. Amino acid sequence of the CD33xCD28 IgG4-scFv$_2$ light and heavy chains.

The amino acid sequence shown in green corresponds to the anti-CD28 (clone E1P2), while the sequence in dark purple represents the anti-CD33-scFv$_2$ (clone SGN33). The IgG4 backbone is highlighted in grey, and the linkers are depicted in black.
