# Supplementary material for: Enhancement of CD117-Targeted Bispecific T-cell Engagement by CD33-Targeted Bispecific T-cell Costimulation in Acute Myeloid Leukemia
Source: Cancer Res Commun. 2026 Apr 27;6(4):946–60. doi: 10.1158/2767-9764.CRC-25-0672 (PMC13114487; doi:10.1158/2767-9764.CRC-25-0672)
Supplement: Supplementary Figure S2 — Figure S2 shows the expression of CD28 on T-cells and CD80/Cd86 on MOLM-14, HL-60 and KASUMI-1 cell lines. [file crc-25-0672_supplementary_figure_s2_suppsf2.pdf]

Supplementary Figure S2

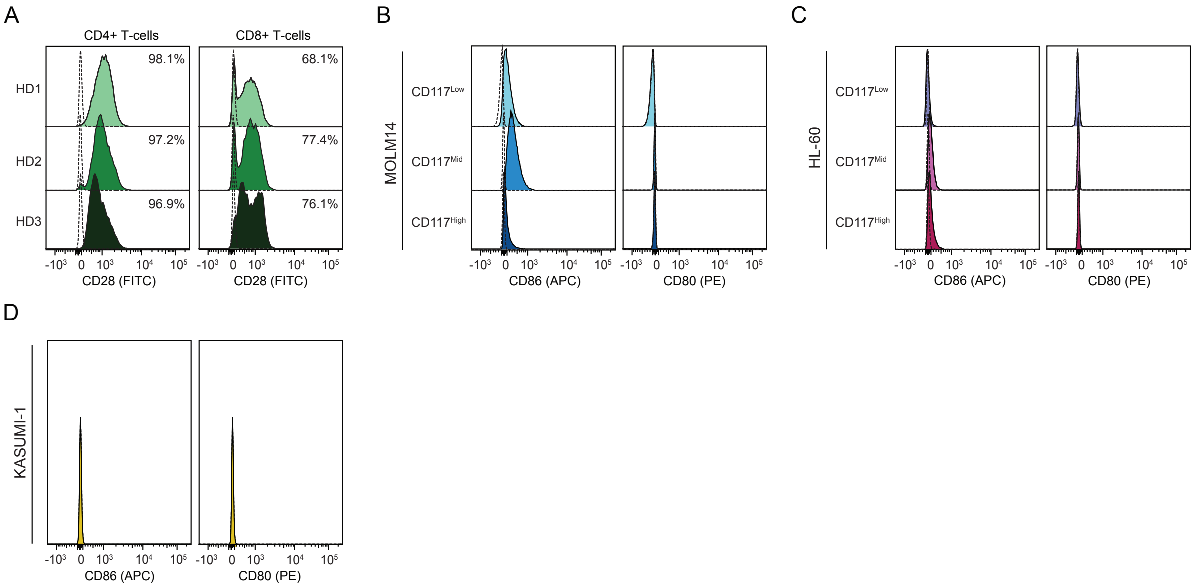

Supplementary Figure S2. Expression of CD28 on T-cells and CD80/CD86 on MOLM-14, HL-60 and KASUMI-1 cell lines. Expression profiles assessed by flow cytometry. **A**. Expression of CD28 on CD4+ (left panel) and CD8+ (right panel) T-cells from 3 healthy donors. **B**. Expression of CD86 and CD80 on MOLM-14 cell line. **C**. Expression of CD86 and CD80 on HL-60. **D.** Expression of CD86 and CD80 on KASUMI-1. Unstained controls represented with the dotted line.
