# Supplementary material for: Enhancement of CD117-Targeted Bispecific T-cell Engagement by CD33-Targeted Bispecific T-cell Costimulation in Acute Myeloid Leukemia
Source: Cancer Res Commun. 2026 Apr 27;6(4):946–60. doi: 10.1158/2767-9764.CRC-25-0672 (PMC13114487; doi:10.1158/2767-9764.CRC-25-0672)
Supplement: Supplementary Figure S3 — Figure S3 shows the dose-dependent effects of CD33xCD28 IgG4-scFv2 on MOLM-14 cell lysis, induced by CD117xCD3 and T-cells. [file crc-25-0672_supplementary_figure_s3_suppsf3.pdf]

Supplementary Figure S3

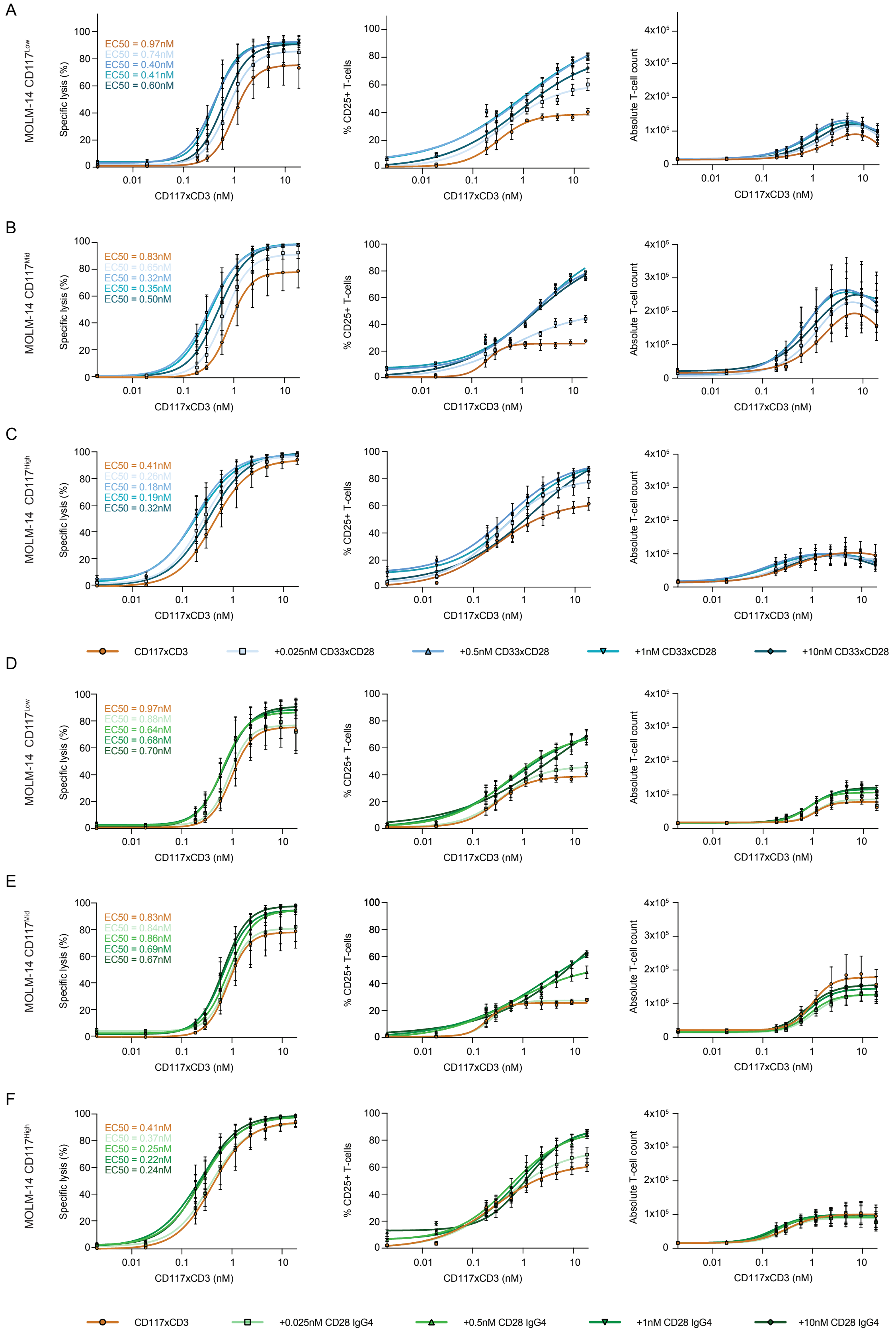

Supplementary Figure S3. CD33xCD28 IgG4-scFv$_2$ dose-dependent effects on MOLM-14 cell lysis, induced by CD117xCD3 and T-cells. MOLM-14 cells

expressing varying levels of CD117 were co-cultured with healthy donor-derived T-cells at an effector-to-target (E:T) ratio of 1:1. CD117xCD3 was added at the indicated concentrations, either alone or in combination with 0.025, 0.5, 1, or 10 nM of CD33xCD28 IgG4-scFv$_2$ (**A-C**) or CD28 IgG4 (**D-F**). After 96 hours, co-cultures were analyzed by flow cytometry. **A-C.** Specific lysis of MOLM-14 CD117^{Low} (**A**), CD117^{Mid} (**B**), and CD117^{High} (**C**) cells (left), percentage of CD25+ T-cells (middle), and T-cell proliferation (right) under the indicated addition of CD33xCD28 IgG4-scFv$_2$. **D-F.** Specific lysis of MOLM-14 CD117^{Low} (**D**), CD117^{Mid} (**E**), and CD117^{High} (**F**) cells (left), percentage of CD25+ T-cells (middle), and T-cell proliferation (right) under the indicated addition of CD28 IgG4. Data represent mean ± SEM from three independent healthy donor-derived T-cell samples, each analyzed in duplicate.
