# Supplementary material for: Enhancement of CD117-Targeted Bispecific T-cell Engagement by CD33-Targeted Bispecific T-cell Costimulation in Acute Myeloid Leukemia
Source: Cancer Res Commun. 2026 Apr 27;6(4):946–60. doi: 10.1158/2767-9764.CRC-25-0672 (PMC13114487; doi:10.1158/2767-9764.CRC-25-0672)
Supplement: Supplementary Figure S4 — Figure S4 shows the dose-dependent effects of CD33xCD28 IgG4-scFv2 on HL-60 cell lysis, induced by CD117xCD3 and T-cells. [file crc-25-0672_supplementary_figure_s4_suppsf4.pdf]

Supplementary Figure S4

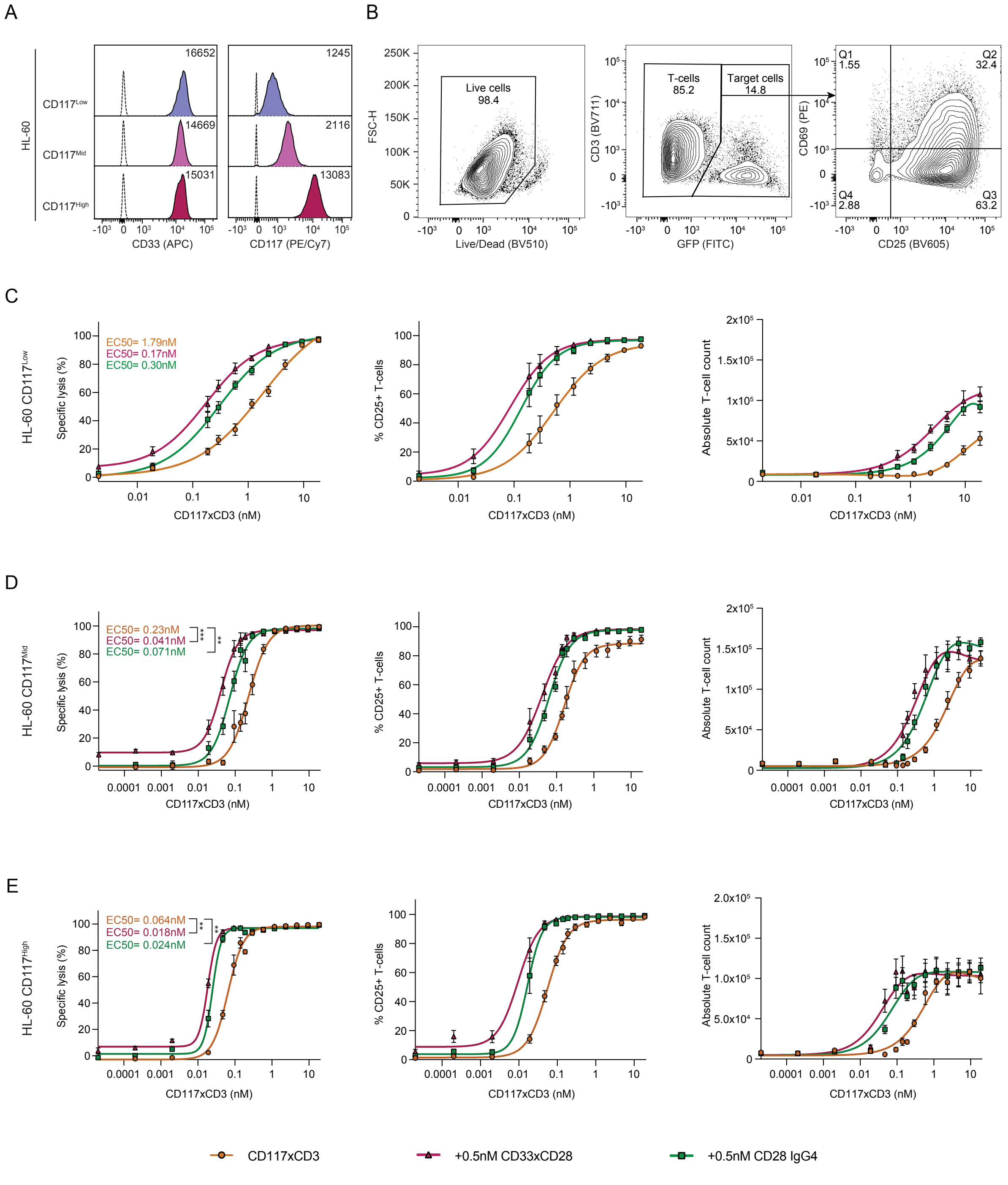

Supplementary Figure S4. CD33xCD28 IgG4-scFv$_2$ dose-dependent effects on HL-60 cell lysis, induced by CD117xCD3 and T-cells. A. Representative histograms of CD117 and CD33 expression on HL-60 AML cells, transduced to express CD117 at low, intermediate and high levels. Unstained control of each cell line shown with dotted line. **B**. Flow cytometry plots with representative gating strategy at 96 hours of co-culture of HL-60 cells with healthy-donor derived T-cells at E:T=1:1 treated with CD117xCD3 and 0.5nM of binder molecules (specific sample: HL-60

CD117^{Mid}GFP$^+$Luc$^+$ with 0.584nM CD117xCD3 and 0.5nM CD28 IgG4). **C-E**. Percentage specific lysis (left), percentage CD25+ T-cells (middle) and T-cell proliferation (right) of HL-60 CD117 low (**C**), mid (**D**) and high (**E**) cells, co-cultured with healthy-donor-derived T-cells at E:T ratio of 1:1 for 96h and indicated concentrations of CD117xCD3, while constant amounts of 0.5nM of either CD33xCD28 IgG4-scFv$_2$ or CD28 IgG4 antibodies were added. Mean ± SEM from three healthy-donor-derived T-cells, each analyzed in duplicates. Statistical analysis for all the graphs conducted using two-way ANOVA against CD117xCD3 treated cells; *$p < 0.05$, **$p < 0.01$, ***$p < 0.001$; ****$p < 0.0001$.
