# Supplementary material for: Enhancement of CD117-Targeted Bispecific T-cell Engagement by CD33-Targeted Bispecific T-cell Costimulation in Acute Myeloid Leukemia
Source: Cancer Res Commun. 2026 Apr 27;6(4):946–60. doi: 10.1158/2767-9764.CRC-25-0672 (PMC13114487; doi:10.1158/2767-9764.CRC-25-0672)
Supplement: Supplementary Figure S6 — Figure S6 shows that T-cells maintain specific lytic activity after repetitive challenge with MOLM-14 CD117High cells. [file crc-25-0672_supplementary_figure_s6_suppsf6.pdf]

Supplementary Figure S6

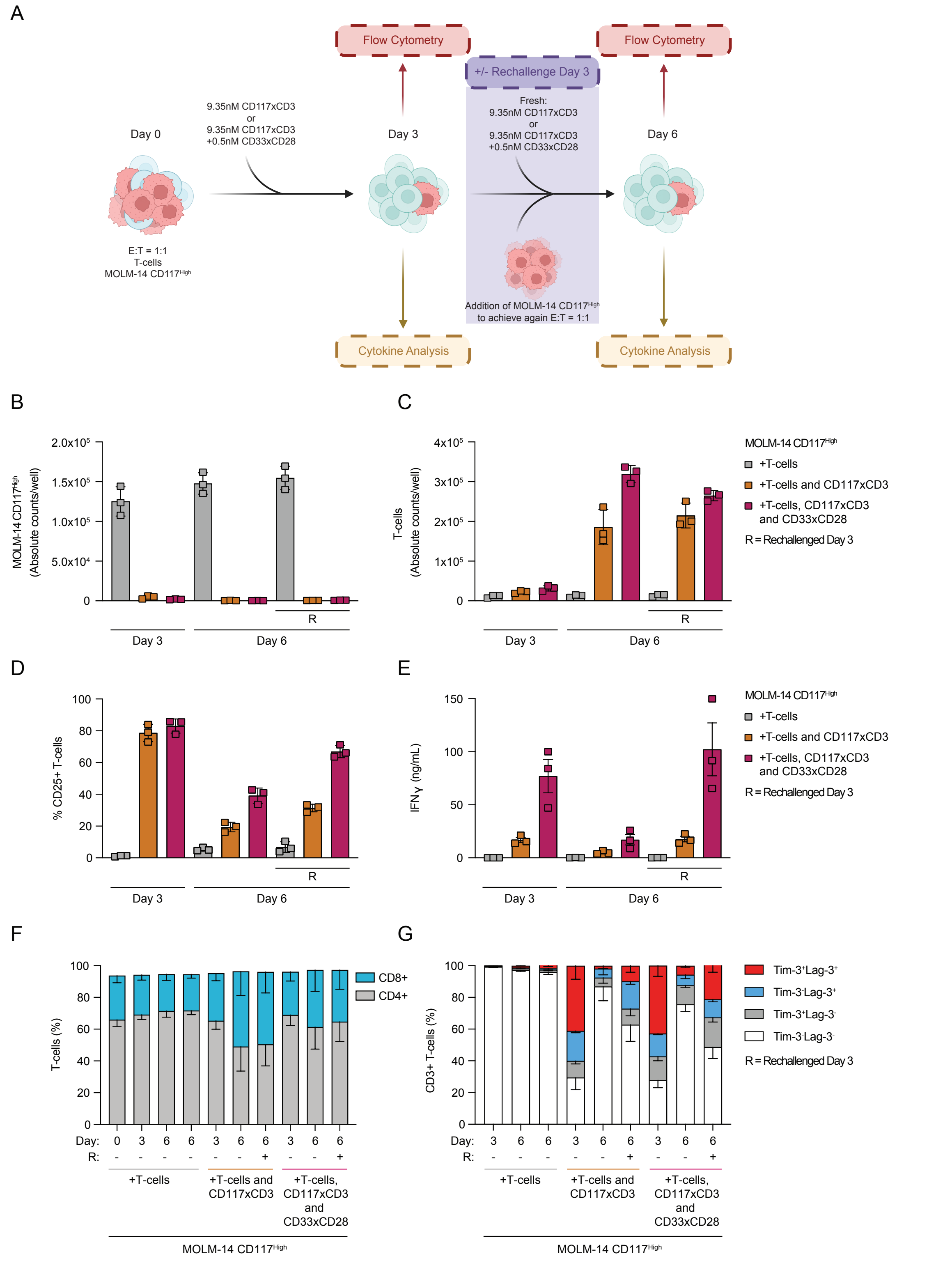

Supplementary Figure S6. T-cells maintain specific lytic activity after repetitive challenge with MOLM-14 CD117^{High} cells. A. Schematic of the experimental workflow (created by Biorender) of the rechallenge experiment. MOLM-14 CD117^{High} cell line was plated with T-cells at an E:T ratio of 1:1. The cells were incubated with

either PBS, 9.35nM CD117xCD3 TCE alone, or in combination with 0.5nM CD33xCD28 IgG4-scFv$_2$. After 3 days, specific lysis was assessed by flow cytometry, and fresh drugs and new MOLM-14 CD117^{High} cells were added to again achieve an E:T ratio of 1:1. After an additional 3 days, flow cytometry and cytokine analysis were performed again. **B-C.** Absolute counts of MOLM-14 CD117^{High} (**B**) and T-cells (**C**) on day 3 and day 6 with or without rechallenge. **D.** Percentage of CD25 positive T-cells. **E.** Interferon-gamma cytokines in the supernatant. **F.** Percentage CD4 and CD8 positive T-cells. **G.** Percentage of TIM-3 and LAG-3 expression on T-cells. Mean ± SEM from three healthy-donor-derived T-cells, each analyzed in duplicates.
