# Supplementary material for: Enhancement of CD117-Targeted Bispecific T-cell Engagement by CD33-Targeted Bispecific T-cell Costimulation in Acute Myeloid Leukemia
Source: Cancer Res Commun. 2026 Apr 27;6(4):946–60. doi: 10.1158/2767-9764.CRC-25-0672 (PMC13114487; doi:10.1158/2767-9764.CRC-25-0672)
Supplement: Supplementary Figure S7 — Figure S7 shows the increased T-cell proliferation at 96h upon addition of CD33xCD28 IgG4-scFv2 to CD117xCD3 TCE in T-cell and primary AML cell co-culture. [file crc-25-0672_supplementary_figure_s7_suppsf7.pdf]

Supplementary Figure S7

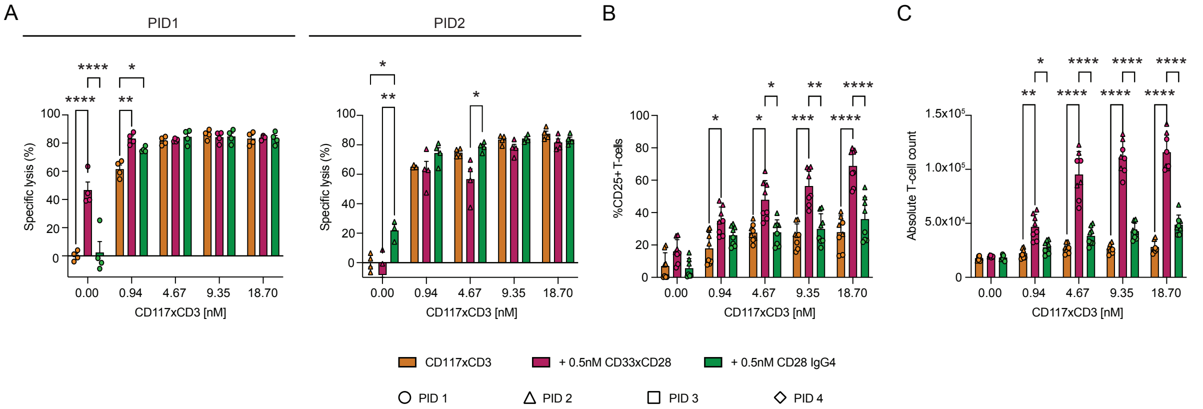

Supplementary Figure S7. Increased T-cell proliferation at 96h upon addition of CD33xCD28 IgG4-scFv$_2$ to CD117xCD3 TCE in T-cell and primary AML cell co-culture. Primary AML samples were co-cultured with healthy donor-derived T-cells at an effector-to-target (E:T) ratio of approximately 1:1. Cells were incubated for 96 hours with CD117xCD3 at the indicated concentrations, in combination with 0.5 nM CD33xCD28 IgG4-scFv$_2$ or 0.5 nM CD28 IgG4. **A.** Percentage specific lysis of PID1 and PID2 at 96h. **B.** Percentage of CD25+ T-cells. **C.** Proliferation of T-cells. Data represent mean ± SEM from two independent healthy donor-derived T cell samples, each analyzed in duplicate. Statistical significance was determined using two-way ANOVA: *$p < 0.05$, **$p < 0.01$, ***$p < 0.001$; ****$p < 0.0001$.
