# Supplementary material for: Enhancement of CD117-Targeted Bispecific T-cell Engagement by CD33-Targeted Bispecific T-cell Costimulation in Acute Myeloid Leukemia
Source: Cancer Res Commun. 2026 Apr 27;6(4):946–60. doi: 10.1158/2767-9764.CRC-25-0672 (PMC13114487; doi:10.1158/2767-9764.CRC-25-0672)
Supplement: Supplementary Table S1 — Table S1 shows the list of antibodies used for flow cytometry. [file crc-25-0672_supplementary_table_s1_suppst1.pdf]

Supplementary Table S1

List of antibodies used for flow cytometry

Target	Flurophore	Clone	Provider	Catalog number, RRID
Live/Dead Zombie Aqua	Brilliant violet 510™	NA	Biolegend	423107
Live/Dead Zombie Violet	eFluor™ 450	NA	Biolegend	423113
Hoechst 33342	eFluor™ 450	NA	Thermo Fischer Scientific	H3570
Goat Anti-human IgG (H+L)	APC	Polyclonal	Invitrogen	A-21445, AB_2535862
Human CD3	APC	UCHT1	Biolegend	300439, AB_2562045
Human CD3	Brilliant violet 711™	UCHT1	Biolegend	300464, AB_2566036
Human CD4	APC-Cyanine7	OKT4	Biolegend	344616, AB_2028483
Human CD4	PE-Cyanine7	SK3	Biolegend	344612, AB_2028479
Human CD8	PE-Cyanine7	SK1	Biolegend	344712, AB_2044008
Human CD8	APC	SK1	Biolegend	344722, AB_2075388
Human CD28	FITC	CD28.2	Biolegend	302906, AB_314308
Human CD69	PE	FN50	Biolegend	985202, AB_2924641
Human CD25	Brilliant violet 605™	BC96	Biolegend	302632, AB_11218989
Human CD25	APC-Cyanine7	BC96	Biolegend	302614, AB_314284
Human CD33	APC	WM53	Biolegend	983902, AB_2810824
Human CD33	BV711	WM53	Biolegend	303423, AB_2565774
Human CD117	PE-Cyanine7	104D2	Thermo Fisher Scientific	25-1178-42, AB_10718535
Human CD80	PE	L307.4	BD Bioscience	560925, AB_396606
Human CD86	APC	2331	BD Bioscience	560956, AB_398608
Human CD45	eFluor™ 450	HI30	Thermo Fisher Scientific	48-0461-82, AB_2574019
Human CD19	PE	HIB19	Invitrogen	12-0199-42, AB_1834376
Human LAG-3	BV605	11C3C65	Biolegend	369324, AB_2721541
Human TIM-3	PE	F38-2E2	eBiosciences	12-3109-42, AB_2572605
Mouse CD45	PerCP-Cyanine5.5	30-F11	Biolegend	103132, AB_893340
