# Supplementary material for: Enhancement of CD117-Targeted Bispecific T-cell Engagement by CD33-Targeted Bispecific T-cell Costimulation in Acute Myeloid Leukemia
Source: Cancer Res Commun. 2026 Apr 27;6(4):946–60. doi: 10.1158/2767-9764.CRC-25-0672 (PMC13114487; doi:10.1158/2767-9764.CRC-25-0672)
Supplement: Supplementary Table S2 — Table S2 shows the list of primary AML patient samples and their genetic characteristics. [file crc-25-0672_supplementary_table_s2_suppst2.pdf]

Supplementary Table S2. List of primary AML patient samples and their genetic characteristics

#	Age	Sex	Diagnosis	Gene mutations	FISH	Karyotype
1	49	M	AML	*CEBPA* (monoallelic)	n/a	46, XY
2	54	F	AML	*CBFB-MYH1, FLT3, KIT*	CBFB-MYH11 [t(16;16)]	46, XX, t(16,16) (p13; q22) [19]/46, sl, del (7)(q31)[1]
3	68	F	AML	*NPM1, TP53, FTL3 ITD*	n/a	46, XX
4	50	F	AML	*NPM1, DNMT3A, NRAS*	n/a	46, XX
